# Rapid SARS-CoV-2 Seroprevalence Survey in Central and Western Divisions of Fiji, 2021

**DOI:** 10.3201/eid2901.221514

**Published:** 2023-01

**Authors:** Stephanie J. Curtis, Abdul W. Shah, Ana Ratu, Donald J. Wilson, Philip C. Hill, Phil Hulcome, Cathy Gaylard, Daniel Faktaufon, Talica Cabemaiwai, Isireli Rabukawaqa, Tevita Qoriniasi, Akesh Narayan, Susana Nakalevu, Pablo Romakin, Jemesa Tudravu, James Fong, Nick Walsh

**Affiliations:** National Critical Care and Trauma Response Centre, Darwin, Northern Territory, Australia (S.J. Curtis, C. Gaylard, N. Walsh);; Ministry of Health and Medical Services, Suva, Fiji (A.W. Shah, D. Faktaufon, T. Cabemaiwai, I. Rabukawaqa, T. Qoriniasi, A. Narayan, S. Nakalevu, P. Romakin, J. Tudravu, J. Fong);; Fiji National University, Suva (A. Ratu, D.J. Wilson, P.C. Hill);; University of Otago, Dunedin, New Zealand (P.C. Hill); Fiji Program Support Facility, Suva (P. Hulcome)

**Keywords:** COVID-19, respiratory infections, severe acute respiratory syndrome coronavirus 2, SARS-CoV-2, SARS, coronavirus disease, zoonoses, viruses, coronavirus, antibodies, seroepidemiologic studies, respiratory infections, disease outbreak, Pacific Islands, Fiji

## Abstract

During November–December 2021, we performed a SARS-CoV-2 seroprevalence survey in Central and Western Divisions of Fiji. A total of 539 participants 8–70 years of age were 95.5% (95% CI 93.4%–97.1%) seropositive, indicating high community levels of immunity. Seroprevalence studies can inform public health responses to emerging SARS-CoV-2 variants.

In Fiji, the SARS-CoV-2 Delta variant wave occurred in a largely unvaccinated and nonimmune population during April–November 2021 (*1*). A risk-based COVID-19 vaccine rollout strategy commenced in March 2021, and, by late November 2021, a total of 90.6% of persons >18 years of age had received 2 vaccine doses and 97.3% had received 1 dose (*1*). Because of the high vaccination coverage, the government of Fiji had planned to reopen international borders by early December 2021.

Serosurveillance is a fundamental component of public health response to disease. Estimating disease epidemiology, including population immunity, by using serosurveillance can inform government policy. In November 2021, no serosurveillance data were available from any Pacific Island country or territory, and data from this region remain sparse (*2*). To inform public health decisions on the safe opening of international borders, we performed a rapid operational estimate of SARS-CoV-2 seroprevalence in Central and Western Divisions of Fiji.

We conducted a single cross-sectional serosurvey during November 24–December 1, 2021. The study population comprised persons deemed at higher risk for SARS-CoV-2 infection who were 8–70 years of age and located in Central and Western Divisions of Fiji. Persons at higher risk of infection included those who worked in healthcare, tourism, hospitality, retail, transportation, and education industries and primary or secondary school students. We used convenience sampling through nonrandom selection of study participants. The government of Fiji identified recruitment sites for persons at high risk for COVID-19 and directly invited those sites to identify potential study participants. We aimed to recruit 550 participants, including at least 50 unvaccinated persons, who were a nationally representative sample of sex and age within the community (*3*).

Field teams were trained in data collection protocols that aligned with the World Health Organization UNITY protocol (*4*). We approached potential participants at recruitment sites and administered a brief questionnaire (Appendix) and collected blood samples after informed consent was obtained. We tested serum samples for IgG against the receptor-binding domain of SARS-CoV-2 by using the SARS-CoV-2 Ab ELISA (Beijing Wantai Biologic, https://www.ystwt.cn), which has 96.7% sensitivity and 97.5% specificity across Alpha and Beta variants. 

We merged questionnaire and laboratory data, summarized covariates, and estimated seroprevalence with 95% CIs. We performed analyses using R version 4.0.2 (The R Project for Statistical Computing, https://www.r-project.org). The Fiji National Human Research Ethics Committee reviewed all study materials and provided a letter of approval; however, formal ethics approval was not required.

We recruited 539 participants who were 30 +14 years of age (mean +SD); 47.3% (255/539) were male and 52.7% (284/539) female (Table). Most participants had received 2 doses of a COVID-19 vaccine (443/539, 82.2%), including 94.9% (463/488) of participants who were eligible for vaccination during the survey period. Overall seroprevalence was 95.5% (95% CI 93.4%–97.1%). Of those who had not received a COVID-19 vaccine, 75% (58/76) were seropositive, including 70% (36/51) of children 8–11 years of age who were not eligible for vaccination during the survey period, indicating serum antibodies resulted from acquired infections.

**Table:** Participant characteristics from a rapid SARS-CoV-2 seroprevalence survey in Central and Western Divisions of Fiji, November–December 2021

Characteristics	No. (%)*	No. seropositive†	Seroprevalence, % (95% CI)
Total	539	515	95.5 (93.4–97.1)
Sex			
M	255 (47.3)	246	96.5 (93.2–98.3)
F	284 (52.7)	269	94.7 (91.3–97.0)
Age, years			
8–11	51 (9.5)	36	70.6 (56.0–82.1)
12–17	73 (13.5)	66	90.4 (80.7–95.7)
18–29	132 (24.5)	132	100 (96.5–100)
30–39	143 (26.5)	141	98.6 (94.5–99.8)
40–49	96 (17.8)	96	100 (95.2–100)
>50	44 (8.2)	44	100 (90.0–100)
Ethnicity			
I-Taukei	269 (49.9)	259	96.3 (93.1–98.1)
Fijian of Indian descent	249 (46.2)	236	94.8 (91.0–97.1)
Fijian of other descent	21 (3.9)	20	95.2 (74.1–99.8)
Occupation industry			
Healthcare worker	158 (29.3)	157	99.4 (96.0–99.9)
Student	142 (26.3)	120	84.5 (77.3–89.8)
Transportation	94 (17.4)	94	100 (95.1–100)
Hotel	69 (12.8)	69	100 (93.4–100)
Education	40 (7.4)	39	97.5 (85.7–99.9)
Hospitality	36 (6.7)	36	100 (88.0–100)
COVID-19 vaccine status			
None	76 (14.1)	58	76.3 (64.9–85.0)
1 dose	20 (3.7)	16	80.0 (55.7–93.4)
2 doses	443 (82.2)	441	99.5 (98.2–99.9)
Self-reported previous COVID-19			
No	410 (76.1)	387	94.4 (91.6–96.3)
Yes	117 (21.7)	117	100 (96.0–100)
Unknown	12 (2.2)	11	91.7 (59.7–99.6)

*Percentage of total. †Number seropositive for SARS-CoV-2.

We demonstrated a high level of immunity in participants >18 years of age in Central and Western Divisions of Fiji during late November and early December 2021. The results provided reassurance that international borders could open to fully vaccinated international travelers, without the necessity of previous hotel quarantine requirements, and relative safety of persons at higher risk for infection could be maintained. Our results highlighted a gap in pediatric vaccine coverage that included children 12–17 years of age who were eligible for vaccination during this investigation and were subsequently prioritized for vaccination by the government of Fiji.

The first limitation of our study is that direct extrapolation of the results to the general public is problematic because we conducted convenience sampling of the study population; however, the Central and Western Divisions account for about two thirds of the population of Fiji and were chosen because of resource availability. Second, our IgG measurements did not differentiate between immunity acquired by vaccination or infection or provide evidence of protection or population risk, particularly for severe COVID-19 (*5*). Hybrid immunity in our study population was likely high because of the recent national Delta variant outbreak and COVID-19 vaccination program (*6*). Third, our investigation occurred before the Omicron variant wave, which resulted in ≈12,000 notified COVID-19 cases during December 2021–February 2022 (*2*). However, the lower Omicron case notifications compared with the previous Delta wave of ≈52,000 cases support the likelihood that high population-level immunity attenuated illness from the highly transmissible Omicron variant when international borders reopened (*7*). Overall, further nationwide seroprevalence studies can inform the public health response as new COVID-19 variants emerge and demonstrate how population immunity might wane in the absence of continued COVID-19 vaccination efforts (*8*–*10*).

Appendix Additional information for rapid SARS-CoV-2 seroprevalence survey in Central and Western Divisions of Fiji, 2021.
