# Supplementary material for: Rapid SARS-CoV-2 Seroprevalence Survey in Central and Western Divisions of Fiji, 2021
Source: Emerg Infect Dis. 2023 Jan;29(1):226–8. doi: 10.3201/eid2901.221514 (PMC9796197; doi:10.3201/eid2901.221514)
Supplement: Appendix — Additional information for rapid SARS-CoV-2 seroprevalence survey in Central and Western Divisions of Fiji, 2021. [file 22-1514-Techapp-s1.pdf]

Article DOI: https://doi.org/10.3201/eid2901.221514

Rapid SARS-CoV-2 Seroprevalence Survey in Central and Western Divisions of Fiji, 2021

Appendix

We used the following participant questionnaire to conduct a rapid SARS-CoV-2 seroprevalence survey in Central and Western Divisions of Fiji during November 24–December 1, 2021.

Unique ID	
Date of interview	
Participant parent/guardian full name (if applicable)	
Participant full name	
Please take a photo and upload the informed consent form.	
Would you like a copy of the study results emailed to you?	□ Yes □ No
Email or phone number	
Sex	□ Male<br>□ Female
Date of birth	
Ethnicity	□ I-Taukei<br>□ Fijian of Indian Descent<br>□ Fijian of Other Descent
Occupation industry	□ Healthcare worker (clinical)<br>□ Healthcare worker (public health)<br>□ Healthcare worker (administrative)<br>□ Education<br>□ Student<br>□ Transport<br>□ Hospitality<br>□ Hotels

	□ Other
Are you vaccinated against COVID-19?	□ No<br>□ Yes, one dose<br>□ Yes, fully vaccinated (two doses)<br>□ Yes, fully vaccinated (three doses)
If yes to any of the above, what is the estimate date of most recent vaccine?	
What brand of vaccine did you receive?	□ AstraZeneca<br>□ Moderna<br>□ Pfizer<br>□ Unknown<br>□ Other
If other, what vaccine brand?	
In the preceding one month, have you had any of the following symptoms suggestive of COVID-19?	For each tick either: Yes, No, Unknown<br>□ Fever (≥38 °C) or history of fever<br>□ Sore throat<br>□ Runny nose (rhinorrea)<br>□ Cough<br>□ Shortness of breath (dyspnea)<br>□ Other respiratory symptoms<br>□ Chills<br>□ Vomiting<br>□ Nausea<br>□ Diarrhea<br>□ Headache<br>□ Rash<br>□ Conjunctivitis<br>□ Muscle aches<br>□ Joint ache(myalgia)<br>□ Loss of appetite<br>□ Loss of smell (anosmia)<br>□ Loss of taste (ageusia)<br>□ Nose bleed<br>□ Fatigue<br>□ Seizures<br>□ Altered consciousness<br>□ Other neurological signs<br>□ Other symptoms
In the preceding six months, have you had any of the following symptoms suggestive of COVID-19?	For each tick either: Yes, No, Unknown<br>□ Fever (≥38 °C) or history of fever<br>□ Sore throat<br>□ Runny nose (rhinorrea)

	□ Cough<br>□ Shortness of breath (dyspnea)<br>□ Other respiratory symptoms<br>□ Chills<br>□ Vomiting<br>□ Nausea<br>□ Diarrhea<br>□ Headache<br>□ Rash<br>□ Conjunctivitis<br>□ Muscle aches<br>□ Joint ache(myalgia)<br>□ Loss of appetite<br>□ Loss of smell (anosmia)<br>□ Loss of taste (ageusia)<br>□ Nose bleed<br>□ Fatigue<br>□ Seizures<br>□ Altered consciousness<br>□ Other neurological signs<br>□ Other symptoms
In 2021, have you been in contact with anyone who tested positive for COVID-19?	□ Yes<br>□ No<br>□ Unknown
If yes to above, what was the estimated date of last contact?	
In 2021, did you live with anyone who tested positive for COVID-19?	□ Yes<br>□ No<br>□ Unknown
If yes to above, what was the estimated date of last contact?	
In 2021, did you work with anyone who tested positive for COVID-19?	□ Yes<br>□ No<br>□ Unknown
If yes to above, what was the estimated date of last contact?	
In 2021, did you test positive for COVID-19?	□ Yes<br>□ No<br>□ Unknown

If yes to above, what month did you test positive?	□ January<br>□ February<br>□ March<br>□ April<br>□ May<br>□ June<br>□ July<br>□ August<br>□ September<br>□ October<br>□ November<br>□ December
In what division was this participant recruited?	□ Western<br>□ Central
